# Supplementary material for: Different Infectivity of Mediterranean and Southern Asian Tomato Leaf Curl New Delhi Virus Isolates in Cucurbit Crops
Source: Plants (Basel). 2022 Mar 6;11(5):704. doi: 10.3390/plants11050704 (PMC8912351; doi:10.3390/plants11050704)
Supplement: Supplementary file 1 [file plants-11-00704-s001.zip › plants-1607975-supplementary.pdf]

Table S1: Primer sets for infectious clone construction of ToLCNDV-ES.

Primer name	Sequence (5'-3')	Target size
I-A-1-F	AAGCTTAAAACGTGTCGTTTCGATCTGG	725bp
I-A-1-R	CTCGAGTAACATCACTAACACAC	
I-A-2-F	CTCGAGGCACCGGACTCAC	2292bp
I-A-2-R	ACTAGTGTTTGTGGATCCAAACTTGGTGAG	
I-B-1-F	AAGCTTTTAGGGAGCGCAGCGACAC	2084bp
I-B-1-R	GGTACCCTATATGGCTATAGGT	
I-B-2-F	GGTACCCTTAACGATCTTGAAC	1586bp
I-B-2-R	ACTAGTCTACAAAAGATAACGAATGGCAAAT	

Table S2: Primer sets for infectious clone construction of ToLCNDV-India.

Primer name	Sequence (5'-3')	Target size
Pa-A-1-F	AAGCTTCCATAAAACTTGTCGTTTCGATC	1336bp
Pa-A-1-R	GGTACCTAAATATGCTTGGTGTA	
Pa-A-2-F	GGTACCTAAGGACCTGCGT	1687bp
Pa-A-2-R	ACTAGTGTTTGTGGATCCGAACTTGGT	
Pa-B-1-F	AAGCTTATTGGCCGCGCAGCGGA	1248bp
Pa-B-1-R	CATATGACGTCACGCATCGAT	
Pa-B-2-F	CATATGTATCATATCCTGCAAAGAC	2432bp
Pa-B-2-R	ACTAGTTTAGCTGGGATAAGAAATGTGTG	
